# Dynamic Mechanical and Charlesby-Pinner Analyses of Radiation Cross-Linked Ethylene-Vinyl Acetate Copolymer (EVA)

**DOI:** 10.3390/molecules30071485

**Published:** 2025-03-27

**Authors:** Anna Svarcova, Petr Svoboda

**Affiliations:** Department of Polymer Engineering, Faculty of Technology, Tomas Bata University in Zlin, Vavreckova 5669, 76001 Zlin, Czech Republic; a_svarcova@utb.cz

**Keywords:** ethylene vinyl acetate, electron beam irradiation, cross-linking, crystallinity, frequency sweep, gel content

## Abstract

The properties of EVA copolymers with various vinyl acetate (VA) contents were compared, with EVA 206 (6 wt.% VA) and EVA 212 (12 wt.% VA) having the same melt flow indices of 2 g/10 min. The impact of electron irradiation at levels of 60, 120, and 180 kGy was studied. Four testing methods were employed as follows: wide-angle X-ray diffraction (WAXD); differential scanning calorimetry (DSC); dynamic mechanical analysis (DMA), using a high-temperature frequency sweep at 150 °C; and gel content analysis. The amount of crystalline phase was determined by WAXD and DSC. Copolymers with a higher VA content (EVA 212) had lower crystallinity. The increase in the amorphous phase allows for the greater movement of radicals, enabling them to react and form cross-links. The effects of the VA content, radiation dose, and frequency on dynamic mechanical properties were investigated by DMA. The DMA analysis focused on the shear storage modulus $G^{\prime }$, damping factor tanδ, and complex viscosity $\eta^{*}$. After irradiation, the damping factor tanδ decreased with an increasing VA content, indicating improved elasticity and a higher degree of cross-linking. A gel content analysis was used to calculate the parameters of the Charlesby-Pinner and Charlesby–Rosiak equations, which help with the determination of the relationship between cross-linking and chain scission. The ratio of cross-linking to scission G(X)/G(S) was higher for the EVA with a higher VA content (EVA 212). Due to a higher VA content (12 wt.%), EVA 212 exhibits more efficient network formation.

## 1. Introduction

Exposure of polymers to electron beams triggers radiation-induced reactions, resulting in alterations due to radiation cross-linking. High-energy electron irradiation leads to chemical changes in macromolecules. Some polymers exhibit radiation resistance when cross-linking prevails in degradation [1]. When some polymers are exposed to fast electrons, their long molecular chains become ionized and connect, forming a cross-linked polymer matrix. The cross-linked matrix has better properties than the original material, including improved heat resistance, reduced distortion at high temperatures, better creep resistance, and enhanced impact strength. Cross-linking also helps the material retain its shape under pressure. By linking the polymer chains, their movement is restricted, turning a liquid polymer exposed to high temperatures above melting point into a solid or gel. Cross-linked polymers have a higher molecular mass, making them strong, heat-resistant, and durable against wear and solvents. These properties make cross-linked polymers useful in areas like solid-phase synthesis, extraction processes, and biomedical applications due to their improved insolubility, mechanical strength, stiffness, and rigidity [2].

Ethylene copolymer resins form a foundation for numerous radiation cross-linked applications, including wire and cable insulation, jacketing, and heat-shrinkable products based on polyolefins. These resins often incorporate ethylene-vinyl acetate (EVA), ethylene butyl acrylate (EBA), and ethylene/methacrylic acid resin (EMA) as additives, modifiers, or cross-linking agents [3]. Irradiation of EVA can generate free radicals at C-H bonds, and these, along with radicals formed by bond dissociation (C-H, C-O-C, C-C, or -C=C-), facilitate cross-linking and chain branching. Ionizing radiation directly interacts with the molecular structure to produce free radicals and other species. Polymers exhibit varying responses to radiation, quantifiable by cross-linking and chain scission [4]. Vinyl polymer degradation typically involves a C-C bond fracture, while cross-linking is associated with C-H bond breakage [5]. Electron beam irradiation from accelerators primarily induces polymer cross-linking, although some chain scission may also occur [1].

Ethylene-vinyl acetate (EVA) is a random copolymer of ethylene and vinyl acetate (VA) repeating units, exhibiting VA content-dependent properties that are influenced by crystallinity. VA content manipulation allows for control of EVA crystallinity, a subject of extensive research. Sharif et al. have explored the impact of radiation on LDPE/EVA blends, revealing a higher gel content (an insoluble fraction after high-temperature solvent exposure) in pure EVA due to an increased amorphous phase, which is the primary site of radiation cross-linking in polyethylene [6]. Abd El-Rehim and El-Nesr have also investigated the effects of ionizing radiation on untreated sulfonated EVA and PE-EVA blends, demonstrating the positive influence of low-dose electron beam irradiation on EVA; the crystallinity decreases with an increase in the VA content in the EVA. The relationship between cross-linking, crystallinity, and the thermal and rheological properties of EVA has been examined, showing a decrease in crystallinity with an increasing VA content [7].

Alothman observed a decrease in the shear storage modulus $G^{\prime }$ with increasing EVA and VA contents and with a rising temperature, while an increasing frequency has the opposite effect [8].

The variability of EVA properties with VA content is attributed to increased polarity and elasticity with a higher VA content [7,8].

While there are papers about electron beam cross-linking of EVA and its blends, to the best of our knowledge, there are no papers about the influence of VA content on electron beam cross-linked high-temperature properties. The standard mechanical test is usually performed at a temperature below the melting point of the crystallites (e.g., at 20 °C). Sethi et al. determined that an EVA addition to PE/EVA blends has been shown to not alter the low-temperature modulus $E^{\prime }$, but decreases it at elevated temperatures. Irradiation increases $E^{\prime }$ across room- and high-temperature ranges, with minimal changes at low temperatures. The tanδ value shows similar trends, decreasing with irradiation at room and high temperatures but remaining consistent at low temperatures [9]. At −100 °C, the polymer is in a glassy state (the molecules are frozen, immobile), while at 25 and 75 °C, the polymer is in a viscoelastic state (the molecules can move, and when exposed to stress, the material stretches). Chemical cross-linking increases the elasticity, reduces the creep, and lowers tanδ. tanδ is a ratio of $G^{\prime }/G^{\prime \prime }$ and during cross-linking, $G^{\prime }$ increases.

In crystalline polymers, crystallites act as physical junctions, forming a maximum molar mass structure through intercrystallite chain cross-linking. A cross-link density at a given irradiation dose is determined by the amorphous content, as crystalline regions are only minimally affected by irradiation [10]. Cross-linking primarily occurs in the amorphous regions and at the surface of crystalline regions [5]. Testing can be performed at temperatures above the melting point when there is no physical cross-linking due to the absence of crystal lamellae.

Radiation cross-linking has been widely investigated for modifying polymer-blend properties in cable insulation applications. Ramachandrand et al. investigated EOC-PDMS blends. Studies have shown improved creep resistance after irradiation, surpassing the peroxide cross-linking in rate and efficiency [11]. Research by Shin et al. on PA6/PP blends with the reactive agent glycidyl methacrylate (GMA) has demonstrated significant increases in the complex viscosity and shear storage modulus after electron beam exposure [12].

This study examines how electron beam irradiation affects the cross-linkability and mechanical properties of ethylene-vinyl acetate (EVA) copolymers. The influence of the VA content (or branching density) on cross-linkability by high-temperature (150 °C) frequency sweep DMA analysis and cross-link density by the gel content was examined. The gel content results were evaluated using the Charlesby–Pinner equation. Sol–gel analysis is a well-established technique for evaluating the unreacted fraction in cross-linked polymers as well as monitoring the curing process in situ [13]. We investigated the effect of the VA content, radiation dose, and frequency on dynamic mechanical properties with a focus on the shear storage modulus $G^{\prime }$, damping factor tanδ, and complex viscosity $\eta^{*}$ of the EVA copolymer.

## 2. Results and Discussion

### 2.1. WAXD Analysis

**Figure 1:**
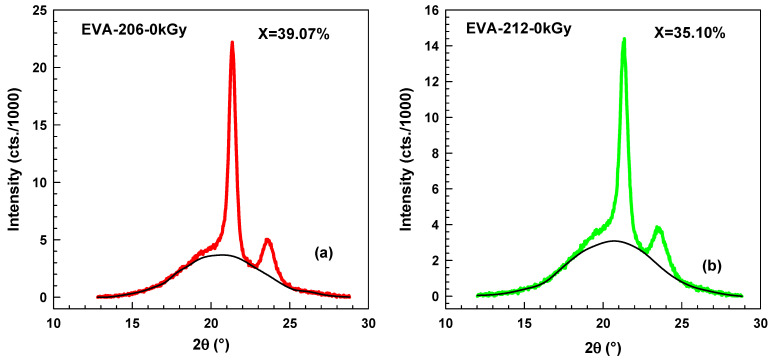
WAXD measurement of two untreated EVA copolymers for crystallinity determination: (**a**) EVA 206–0 kGy and (**b**) EVA 212–0 kGy. The color curves show the original traces, the black curves illustrate the amorphous part.

Initially, we used wide-angle X-ray diffraction (WAXD) to assess the crystallinity of the original copolymers. Figure 1 shows two traces of X-ray scattering; the color curves show the original traces, while the black curves illustrate the amorphous part. EVA 206 had higher crystallinity, or, in other words, EVA 212 contains more amorphous phase.

Table 1 contains the values of crystallinity, evaluated by wide-angle X-ray scattering. The influence of irradiation is extremely small; with an increasing dose, the crystallinity decreased slightly. However, the influence of the VA content is significant; EVA 212 exhibits much lower values.

### 2.2. DSC Analysis

**Figure 2:**
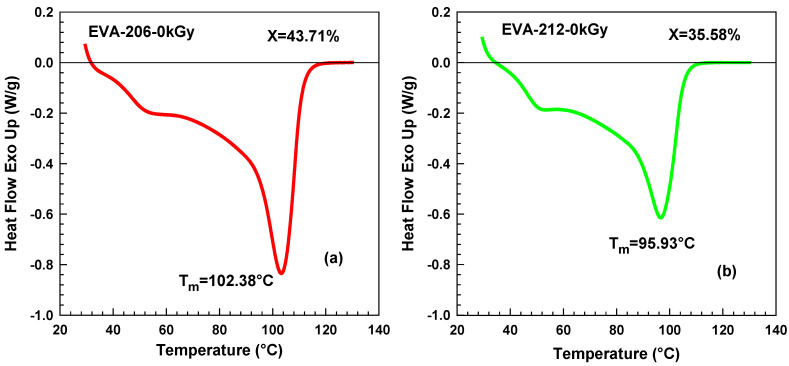
DSC measurements of two untreated EVA copolymers for melting point and crystallinity determination; the curves were normalized for sample size: (**a**) EVA 206–0 kGy and (**b**) EVA 212–0 kGy.

Figure 2 depicts the heat flow curves of the untreated copolymers before any thermal treatment. EVA 206 exhibits a higher melting point and higher crystallinity. The higher VA content hinders crystallization, and therefore, EVA 212 has a lower melting point and lower crystallinity.

**Figure 3:**
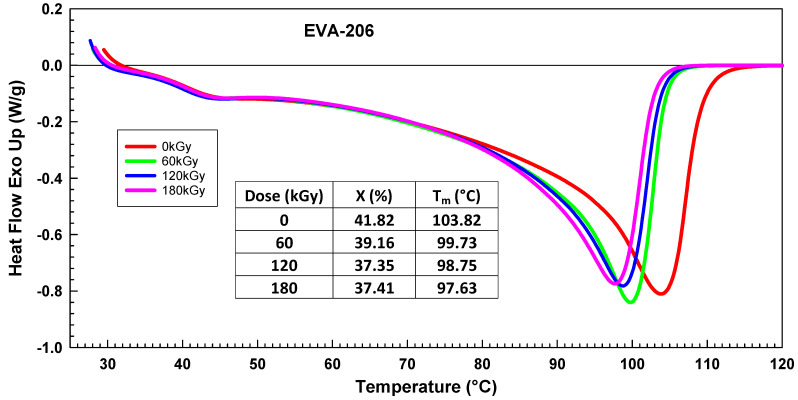
DSC measurements of the melting points and crystallinities of EVA 206 for various irradiation levels after heating to 150 °C at a rate of 10 °C/min followed by cooling at a rate of 5 °C/min.

Figure 3 shows the influence of irradiation on melting point $\left(T_{m}\right)$ and crystallinity (X) for the EVA 206 copolymer. Both $T_{m}$ and X systematically decrease with an increasing irradiation dose. For dose 120 and 180 kGy, the crystallinity is almost the same.

Table 2 contains the melting point and crystallinity from DSC. Data in the first two columns were obtained from the samples directly after irradiation. Data in columns three and four were determined after a very slow cooling rate of 3 °C/min. From the data, it is visible that EVA 212 has lower melting temperatures and also lower crystallinity. The melting point and crystallinity decrease with an increasing dose.

**Figure 4:**
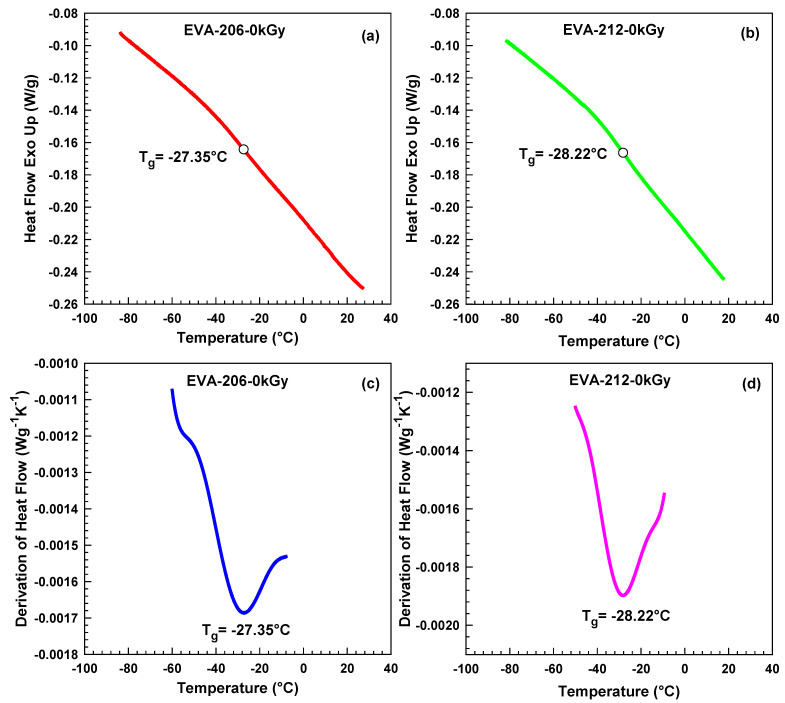
DSC measurement of glass transition temperature $T_{g}$ for untreated EVA copolymers: (**a**) EVA 206–0 kGy original heat flow, (**b**) EVA 212–0 kGy original heat flow, (**c**) EVA 206–0 kGy derivation of heat flow, and (**d**) EVA 212–0 kGy derivation of heat flow.

Between glass transition $T_{g}$ and melting point $T_{m}$, the chains have high mobility, and when exposed to irradiation, the radicals can approach each other, recombine, and form a cross-link. Information on $T_{m}$ and $T_{g}$ is crucial for a discussion of the influence of irradiation on EVA copolymers, and therefore, we included the $T_{g}$ measurement into our study (see Figure 4). Both $T_{g}$ values are very similar, −27.35 °C and −28.22 °C for EVA 206 and EVA 212, respectively. Our $T_{g}$ values seem to fit into the range reported by Carotenuto et al., being −25 to −35 °C [14].

According to many sources, cross-linking occurs predominantly in an amorphous phase. It seems to be the main factor in this paper, with EVA 212 being more amorphous than EVA 206. Let us examine the literature in historical order on this topic. Sawatari and Matsuo, in 1987, studied electron beam cross-linked polyethylene [15]. They concluded that cross-linking in polyethylene occurs preferentially in the amorphous phase. Han et al., in 2004, studied cross-linking and degradation of polypropylene by e-beam irradiation in the presence of trifunctional monomers [16]. They have observed that the cross-linking occurred mainly at the boundary surfaces between crystalline and amorphous regions. They also mention that the degree of cross-linking in PP becomes lower with rising crystallinity. Ries and Pruitt, in 2005, stated that cross-linking by chemical treatment or by irradiation renders covalent bonds between polymer chains in the amorphous phase but definitely not in the crystalline phase [17]. Dias and Silva studied polyethylene foams cross-linked by e-beam and they also concluded that cross-linking happens mainly in amorphous regions [18]. Koo et al. concluded that cross-linking took place mainly in amorphous regions of starch granules, and the crystalline pattern of starch by WAXD remained unchanged [19]. Makuuchi and Cheng wrote about cross-linking in semicrystalline polymers and concluded that cross-linking occurs predominantly in amorphous regions [20]. Gao et al. [21] and Yuan et al. [22] also concluded that a cross-linked network among polymer chains happens mainly within the amorphous region.

**Scheme 1:**
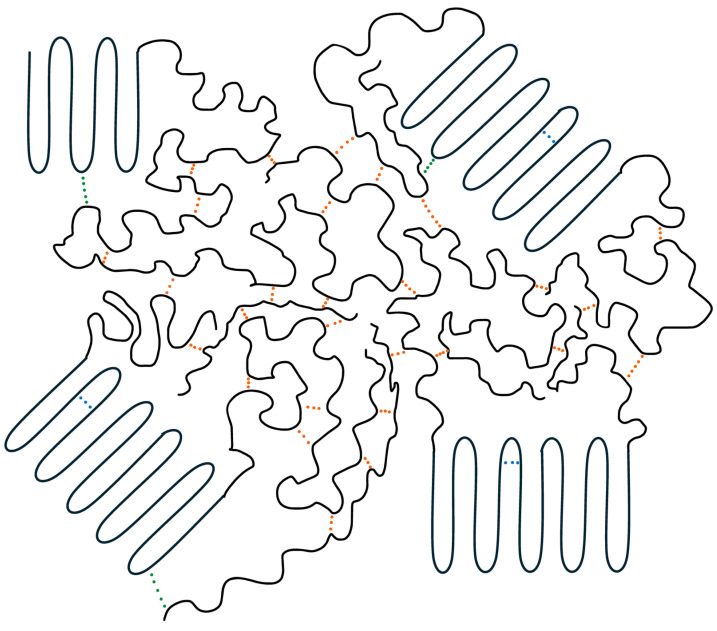
Possible cross-linking mechanisms in semicrystalline polymer containing crystalline and amorphous regions. The blue dots represent the cross-links inside crystalline lamellar regions, the green dots are cross-links between the surface of lamellae and the amorphous chain, and the orange dots are cross-links between the amorphous chains.

Scheme 1 shows the cross-linking possibilities inside semicrystalline EVA. Dots with different colors represent three cross-linking possibilities. The blue dots are the cross-links inside crystalline lamellar regions where the movement of radicals is extremely restricted, and the probability of this cross-linking is very low. The green dots are cross-links between the surface of lamellae and the amorphous chain. The orange dots are cross-links between the amorphous chains that have the highest mobility and radicals that have the highest probability of recombination. The number of these cross-links is most likely the highest.

### 2.3. Frequency Sweep by Dynamic Mechanical Analysis (DMA)

Initially, it is necessary to mention the reason why we have performed the DMA experiments at elevated temperatures. At room temperature, the amorphous chains are held by crystal lamellae, and ross-linking to lower levels is not easily detected for semicrystalline polymers at room temperature. Therefore, our experiments were carried out at an elevated temperature (at 150 °C) above the melting point of EVA (around 100 °C). At an elevated temperature, there are no crystal lamellae, and amorphous chains are held together only by cross-linking. Also, it is necessary to mention that we were not able to measure the original samples (0 kGy) since the material would flow freely from the machine in a vertical arrangement. Only the irradiated samples could be measured.

**Figure 5:**
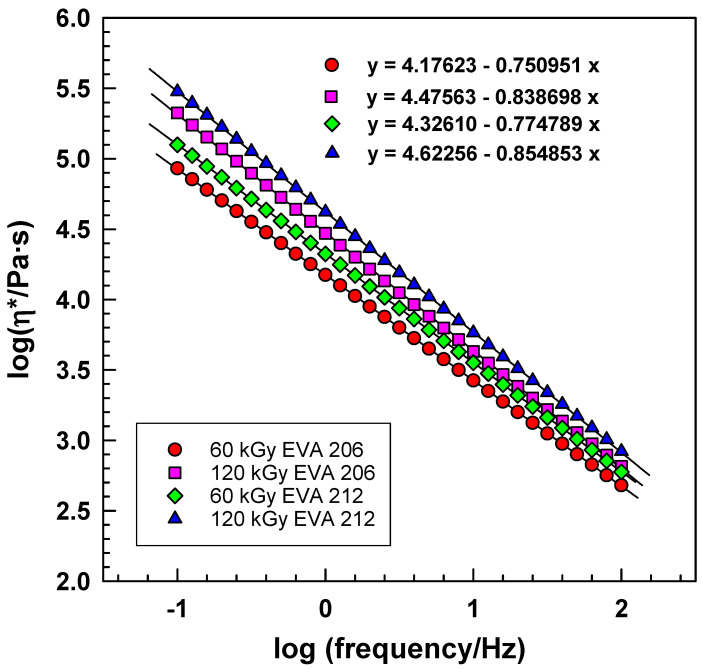
The complex viscosity as a function of the frequency.

Figure 5 shows the complex viscosity $\eta^{*}$ of the samples as a function of frequency at 60 and 120 kGy. The complex viscosity decreases with increasing frequency, indicating pseudoplastic behavior in both irradiated polymers [23]. Generally, copolymers exhibit the rheological behavior of pseudoplastic fluids [24].

The viscosity increases with the increase in the radiation dose. The viscosity increases with the radiation dose, which aligns with the findings of Hui et al. [24]. The complex viscosity sharply decreases with increasing frequency and agrees well with Dutta et al. [25]. This is attributed to the cross-linked structure in irradiated samples, which hinders chain disentanglement [25,26]. The pseudoplasticity arises from the randomly oriented and entangled polymer chains, which align and disentangle under shear, leading to reduced viscosity [23].

Power law fits were applied to the data to determine the shear thinning exponent n [27]. The absolute value of n increases its value with irradiation for both copolymers.

From the studies of complex viscosity by Sung. et al., studies on EVA with and without dicumyl peroxide (DCP) have shown that cross-linking affects the melt-state complex viscosity [28]. For EVA without DCP, n ranged from 0.39 to 0.50, while for EVA with DCP, it ranged from 0.03 to 0.12. Cross-linking with DCP can enhance the melt strength, which is important in applications like foaming [28].

Table 3 illustrates a trend where index n reduces with an increasing dose in irradiated samples. This decline in n signifies the presence of cross-linking in the EVA phase [25,28].

Index n, which represents the slope of the line, exhibits higher absolute values with increasing irradiation doses. For example, in the case of EVA 206, at 60 kGy, n has a value of −0.75095, while for EVA 212 at 120 kGy, it decreases to −0.85485, indicating a steeper decline in viscosity by frequency. This trend is also observed in samples with a higher VA content.

Similarly, the value of k increases with a higher irradiation dose. This trend confirms that more irradiated materials with a higher VA content exhibit greater viscosity, further supporting the impact of the irradiation dose on material properties.

The *k* value that relates to viscosity was found to increase with increasing irradiation for both copolymers. Flow is more restricted in more irradiated samples; it agrees with Hui et al. [24].

A negative index n in a Power law model during irradiated copolymer might signify the prevailing cross-linking effect, which restricts molecular movement or compliance. Cross-linking makes the material stiff and reduces flexibility.

At low frequencies, the viscosity increases significantly due to cross-linking, while at high frequencies, the differences are much smaller. This fact causes an increase in the n value.

Table 3 shows the shear thinning exponent (n) and (k) values from the Power law equation.

**Figure 6:**
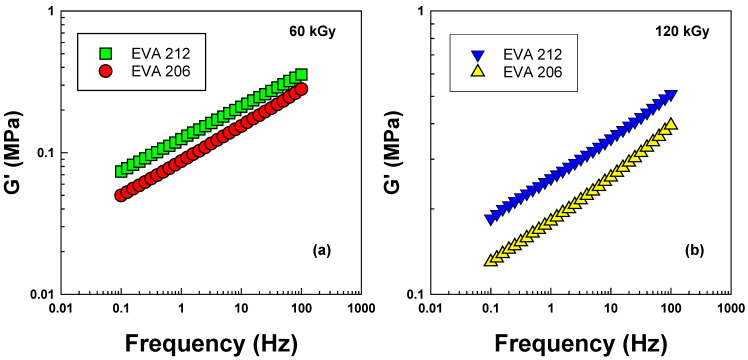
Shear storage modulus versus frequency for EVA 206 and EVA 212: (**a**) 60 kGy and (**b**) 120 kGy.

Figure 6a,b exhibit changes in the shear storage modulus $G^{\prime }$ of the samples EVA 206 and EVA 212 as a function of frequency at 60 kGy and 120 kGy. Across both copolymers and irradiation levels, $G^{\prime }$ demonstrates an increase with frequency. This can be explained by the increased energy input to the polymer chains at higher shear rates, leading to a greater elastic response [11,29,30].

The observed increase in $G^{\prime }$ with frequency reflects the growing restrictions on polymer chain movement. Furthermore, $G^{\prime }$ increases with the increasing radiation dose, which is consistent with the formation of a radiation-induced cross-linked network. The trends in shear storage modulus have confirmed the existence of cross-linking [20].

Comparing Figure 6a,b clearly, higher irradiation causes a higher $G^{\prime }$ shear storage modulus. For both irradiation levels, the EVA 212 copolymer exhibits a higher $G^{\prime }$ shear storage modulus. Additionally, EVA 212 exhibits a higher shear storage modulus than EVA 206 at both irradiation levels. This difference can be attributed to the presence of vinyl groups in the EVA 212 copolymer, which is known to reduce the gelation dose and enhance cross-linking efficiency [31]. The gelation dose is the lowest dose needed to initiate macroscopic gel formation [32]. The concentration of vinyl groups in the amorphous regions increases the probability of a reaction with free radicals, leading to a higher $G^{\prime }$. Furthermore, the shear storage modulus of EVA 206 and EVA 212 copolymers increased with an increasing irradiation dose. Our results are comparable to Shin et al. [12].

Gelation has been theoretically described as a process involving increasing molecular disorder. Percolation theory suggests a relationship as described in Equation (1), where the exponent n is 2/3. However, experimental observations have reported n values near 0.8 for cross-linked PVC, 0.2 for vulcanized PE, and around 0.2 for crystalline polypropylene [33]. The 0.2 value is close to our samples cross-linked by 60 kGy.

Vallés et al. proposed that the gel strength is related to the mobility of chain segments and that the relaxation exponent n can theoretically range from 0 to 1. Specifically, they found that stoichiometrically balanced endlinking reactions in cross-linked silicone and PPO-polyurethane systems yield critical gels with n values of 0.5. Deviations from a stoichiometric balance in these networks resulted in n values between 0.5 and 0.7. Furthermore, cross-linking in the presence of a diluent led to even higher n values. Very low n values were observed in high-molecular-weight PDMS and in crystallizing polypropylene (a physical gel) [34].

The shear storage modulus (*G*′) increases with radiation exposure. This increase is attributed to restricted molecular motion due to cross-linking, which is consistent with the findings of Hui et al. [24]. Moreover, crystallinity increases on exposure to radiation, which also has an effect on the shear storage modulus below melting point [9].

At the gelation point, the moduli are expected to scale with frequency. At the gelation point, the moduli are expected to scale with frequency as described in [33]

(1) $$G^{\prime }\propto G^{\prime \prime }\propto \omega^{n}\;$$

**Figure 7:**
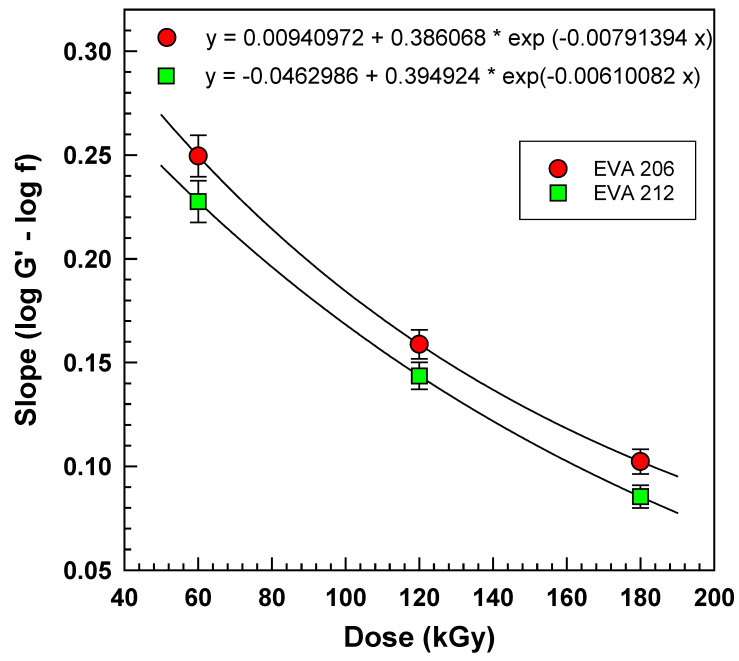
Dependence of slope from (log $G^{\prime }$ − log f) on dose for EVA copolymers and three radiation levels.

Figure 7 illustrates the slope in logarithmic scale and shows the dependence of shear storage modulus on frequency. The use of a logarithmic scale provides insight into a better understanding of a wide range of frequencies, which is typical for rheological analysis.

Figure 7 shows that the slope k, calculated from the dependence of *G*′ on frequency, shows a steeper incline for lower irradiation doses and lower VA content. This indicates that, at initial radiation exposure, the incline of the slope has a higher value for samples with a lower VA content and lower irradiation.

**Figure 8:**
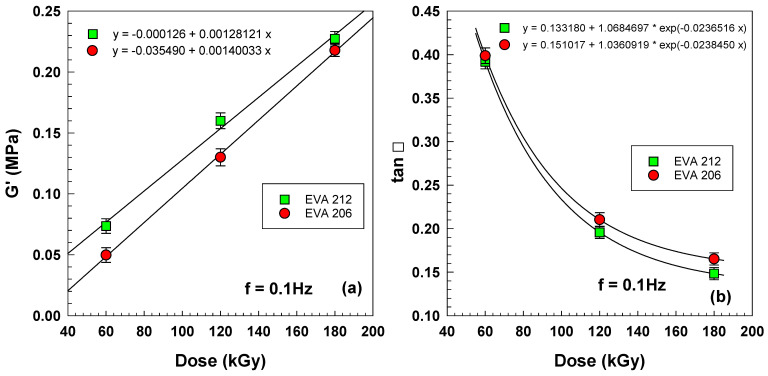
Shear storage modulus versus dose for EVA 206 and EVA 212: (**a**) $G^{\prime }$ and (**b**) tanδ.

Irradiation-induced changes in the properties of viscoelastic materials were monitored by DMA. The shear storage modulus $G^{\prime }$ is related to sample stiffness. Figure 8 shows a decrease in tan δ with increasing radiation exposure. This decline is expected and can be attributed to radiation-induced changes within the material. Concurrently, the shear storage modulus $G^{\prime }$ demonstrates a linear increase with the radiation dose. EVA 212 exhibits higher values of $G^{\prime }$ and lower values of tanδ. Our results agree with Sethi et al. [9].

**Figure 9:**
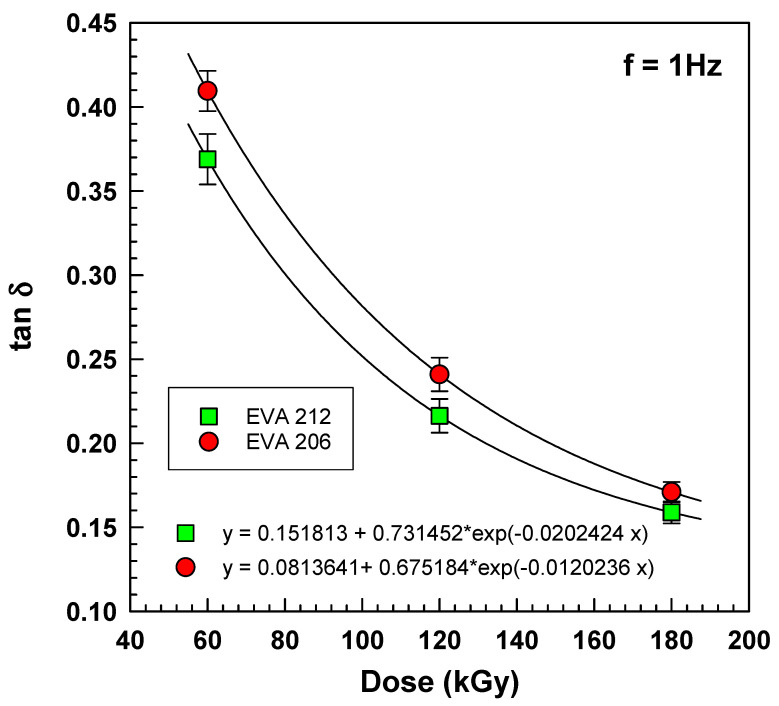
tanδ as a function of dose for EVA 206 and EVA 212 measured at frequency f=1 Hz.

Figure 9 shows a similar exponential decrease in tanδ, as seen in Figure 8b, but this time, it is for the higher frequency f=1 Hz. The difference in tanδ is quite remarkable at the lowest irradiation dose (60 kGy).

**Figure 10:**
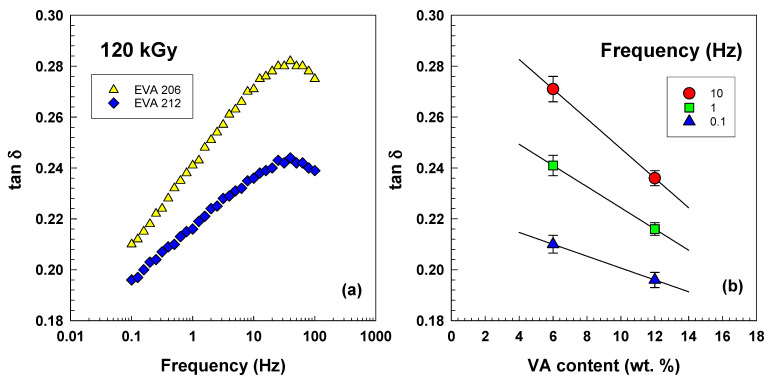
(**a**) tanδ vs. frequency for EVA 206 and EVA 212, and (**b**) tanδ vs. VA content for various frequencies.

Figure 10a shows the tanδ curves for both irradiated copolymers as a function of frequency. Each curve exhibits a maximum. In the tan δ versus frequency plot, a negative slope is characteristic of viscoelastic liquid behavior, whereas a positive slope suggests that the elastic component of the viscoelastic material is dominant. This trend of the tan δ curves is comparable with Durmus et al. [27]. The EVA 212 curve is below EVA 206. Figure 10b shows that tanδ decreases with an increasing VA content. A lower tanδ of EVA 212 means better elasticity and indicates a higher cross-linking level.

Ethylene-vinyl acetate (EVA) is composed of long, linear chains. During exposure to radiation, free radicals are generated within the material. These free radicals can react with each other, leading to the formation of cross-links between the polymer chains.

The Flory–Huggins theory provides a framework for understanding the properties of polymer networks, enabling the measurement of cross-link density. This theory establishes a relationship between the network structure and measurable quantities, allowing for a determination of the average molecular weight between cross-links $M_{C}$. By utilizing the cross-link density $\nu_{d}$ and the specific volume $\bar{\nu }$ of the polymer, $M_{C}$ can be easily calculated [35].

(2) $$M_{C}=\frac{1}{\bar{\nu }\;\nu_{d}}\;$$

The specific volume $\bar{\nu }$ can be expressed as:

(3) $$\bar{\nu }=\frac{1}{\rho }\;$$

where ρ is the density.

Equation (2) can be expressed as:

(4) $$M_{C}=\frac{1}{\bar{\nu }\;\nu_{d}}=\frac{1}{\frac{1}{\rho }\;\nu_{d}}=\frac{\rho }{\;\nu_{d}}$$

Then, the cross-link density can be calculated as:

(5) $$\;\nu_{d}=\frac{\rho }{M_{C}}\;$$

The presence of crystals in EVA at room temperature significantly restricts chain movement, leading to an elevated shear storage modulus that does not accurately reflect the degree of cross-linking. However, when the temperature rises above the melting point, the crystalline structure disappears, allowing for an assessment of the effects of cross-linking and entanglement density. In this melt state, the material becomes completely amorphous, and the loss modulus approaches zero. The shear storage modulus reaches a minimum or plateau, indicating the onset of the rubbery plateau region where the modulus remains relatively stable with further temperature increases.

Studies have revealed a correlation between the molecular weight of cross-links $M_{C}$ and the shear storage modulus $G^{\prime }$. This relationship can be expressed as:

(6) $$G^{\prime }=\frac{\rho RT}{M_{C}}\;$$

where ρ is the density of the amorphous polymer, $R=8.314\;{J\;K}^{-1}{\;\mathrm{mol}}^{-1}$ is the gas constant, and T is the absolute temperature. The DMA collects the shear storage modulus, and the molecular weight between the cross-links $M_{C}$ is then calculated as:

(7) $$M_{C}=\frac{\rho RT}{G^{\prime }}\;$$

Through DMA, it is possible to measure the key properties of polymer networks, such as the molecular weight between cross-links and the cross-link density.

The cross-link density $\nu_{d}$ is then:

(8) $$\;\nu_{d}=\frac{\rho }{M_{C}}=\frac{\rho }{\frac{\rho RT}{G^{\prime }}}$$

Finally, we can calculate the cross-link density $\nu_{d}$ as:

(9) $$\;\nu_{d}=\frac{G^{\prime }}{RT}$$

Table 4 and Table 5 show trends of the increasing shear storage modulus $G^{\prime }$, decreasing molecular weight between cross-links $M_{C}$, and the increasing cross-link density $\nu_{d}$ with an increasing irradiation dose for both EVA copolymers. The copolymer EVA 212, with a higher VA content, had higher values of shear storage modulus $G^{\prime }$ and cross-link density $\nu_{d}$ and lower values of molecular weight between cross-links $M_{C}$ compared to EVA 206. Our results of cross-link density $\nu_{d}$ are comparable to Bandzierz et al. [36].

### 2.4. Gel Content Analysis

While cross-linked polymer networks ideally possess an effectively infinite molecular weight, they often contain a fraction of soluble polymer chains that are not integrated into the network. These non-networked chains can influence the overall properties of the material. This soluble portion is termed the sol fraction, while the insoluble, cross-linked component is referred to as the gel fraction [13].

**Figure 11:**
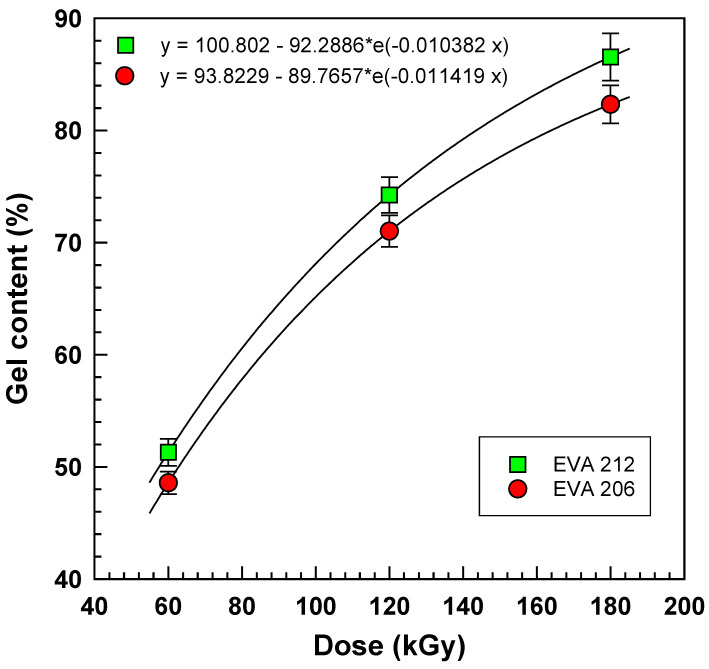
Gel content vs. dose for two radiation cross-linked ethylene-vinyl acetate copolymers.

Figure 11 shows the variations in gel content with increasing doses of electron beam irradiation for two EVA copolymers. The gel fraction of the irradiated samples increases rapidly in the dose range of 60–120 kGy, and then in the range of 120–180 kGy, the increase is rather moderate. Moreover, Figure 11 shows that the amount of gel content is lower for EVA 206. The higher gel content for EVA 212 demonstrates higher cross-linking. Our results are in agreement with Wang et al. [37].

The observed increase in gel content with increasing radiation dose aligns with the work of Datta et al. [38]. Additionally, these findings corroborate the research conducted by Ali and Legocka on the effect of ethylene/methacrylic acid copolymer (EMA) metal salts on the electron beam cross-linking of low-density polyethylene (LDPE). Their study also demonstrated a progressive increase in gel content with increasing irradiation dose across all samples examined [3].

**Figure 12:**
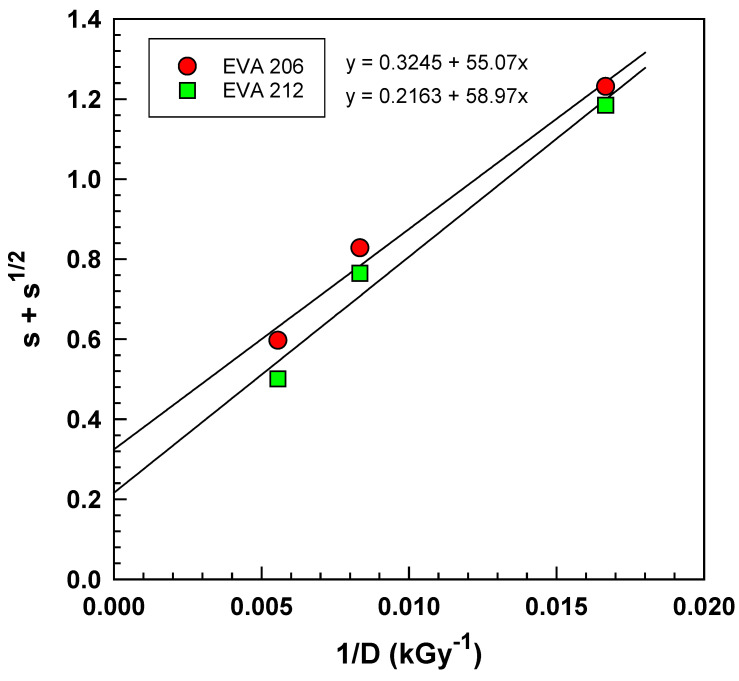
Quantitative Charlesby–Pinner analysis: $s+s^{1/2}$ vs. 1/D of EVA 206 and EVA 212 yielding slope and intercept values used for calculation shown in Supplementary Materials.

Figure 12 presents the sol–gel analysis results, depicting the relationship between (s + s^1/2^) and the inverse of radiation dose (D^−l^)., where D is measured in kGy. The observed linear relationship allowed for the determination of slope values, which were subsequently used in calculations. As expected, the sol fraction decreased while the gel fraction increased with increasing irradiation dose.

The values from the linear regression were used in the calculation of all the Charlesby–Pinner parameters according to Equation (9). Detailed calculations are depicted in the Supplementary Materials.

(10) $$s+\sqrt{s}=\frac{G\left(S\right)}{2G\left(X\right)}+\frac{4.82\times 10^{6}}{G\left(X\right)M_{n}D}\;$$

where s represents the sol fraction, and g is the gel fraction (s+g=1). The radiation chemical yield is expressed using G values, specifically G(X) for cross-linking and G(S) for chain scission. $M_{n}$ is the number-average molecular weight. D is the absorbed radiation dose in kGy. The gel content values in percentage are shown in Figure 11.

To quantify the relative contributions of cross-linking and chain scission, plots of $s+s^{1/2}$ vs. (1/D) were generated using the Charlesby–Pinner equation [4]. Figure 12 displays the results, from which the ratio $p_{0}/q_{0}$ was calculated. This ratio corresponds to the interception of Equation (11) and is directly related to the data presented in Figure 11.

(11) $$s+\sqrt{s}=\frac{p_{0}}{q_{0}}+\frac{1}{q_{0}P_{n}D}$$

where $P_{n}$ is the initial number-averaged degree of polymerization, $q_{0}$ is the cross-linked unit density per unit dose, and $p_{0}$ is the ratio of the main chain breaks to chain units.

The Charlesby–Pinner model was modified by Olejniczak et al. [39], leading to the Charlesby–Rosiak equation:

(12) $$s+\sqrt{s}=\frac{p_{0}}{q_{0}}+\left(2-\frac{p_{0}}{q_{0}}\right)\left(\frac{D_{v}+D_{g}}{D_{v}+D}\right)$$

where $D_{v}$ is the virtual dose, and $D_{g}$ is the gelation dose.

The relationship of 1/D vs. $s+\sqrt{s}$, according to the original Charlesby–Pinner equation, presented in Figure 12, had a deviation from the ideal linearity, caused most likely by the M_w_/M_n_ ratio being different from 2. Therefore, we have used a modified version, presented historically first in 1991 by Olejniczak et al. [39] and then explained in detail in 1998 by Rosiak [40]. This so called Charlesby–Rosiak equation has been used by many authors, e.g., by Furusawa et al. [41], Bandzierz et al. [36], Hidiroglu et al. [42], and many others.

The $D_{v}$ and $D_{g}$ constants can be determined using GelSol95 software [42], or the data can be fitted by a least squares method [41], which was used in this paper.

**Figure 13:**
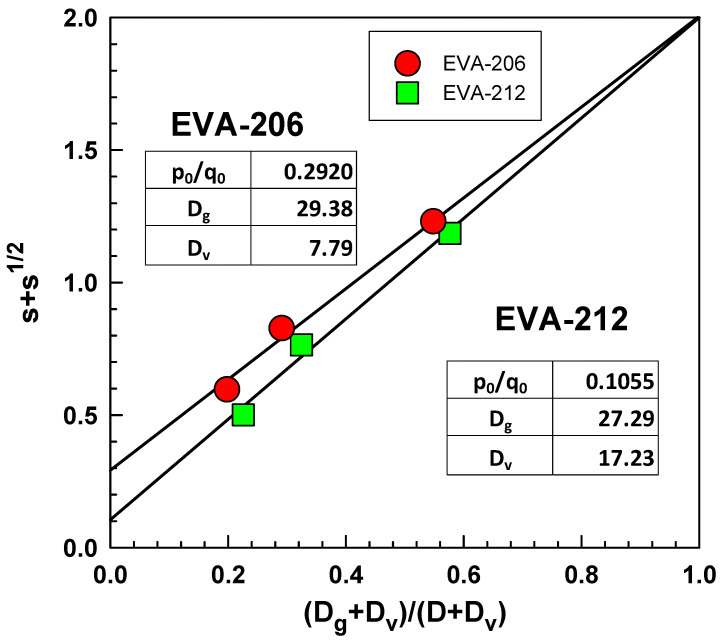
Experimental data of the sol–gel analysis for the EVA copolymers shown in the Charlesby–Rosiak coordinates.

According to the Charlesby–Pinner analysis (Figure 12), the $p_{0}/q_{0}$ values were 0.3246 and 0.2163 according to the Charlesby–Rosiak equation (Figure 13), which were 0.2920 and 0.1055 for EVA 206 and EVA 212, respectively. In both cases, the ratio of the radiation yield of degradation (chain scission) to the radiation yield of cross-linking, represented as the $p_{0}/q_{0}$ value, is smaller for EVA 212. A lower $p_{0}/q_{0}$ value indicates a greater tendency for cross-linking over chain scission.

**Figure 14:**
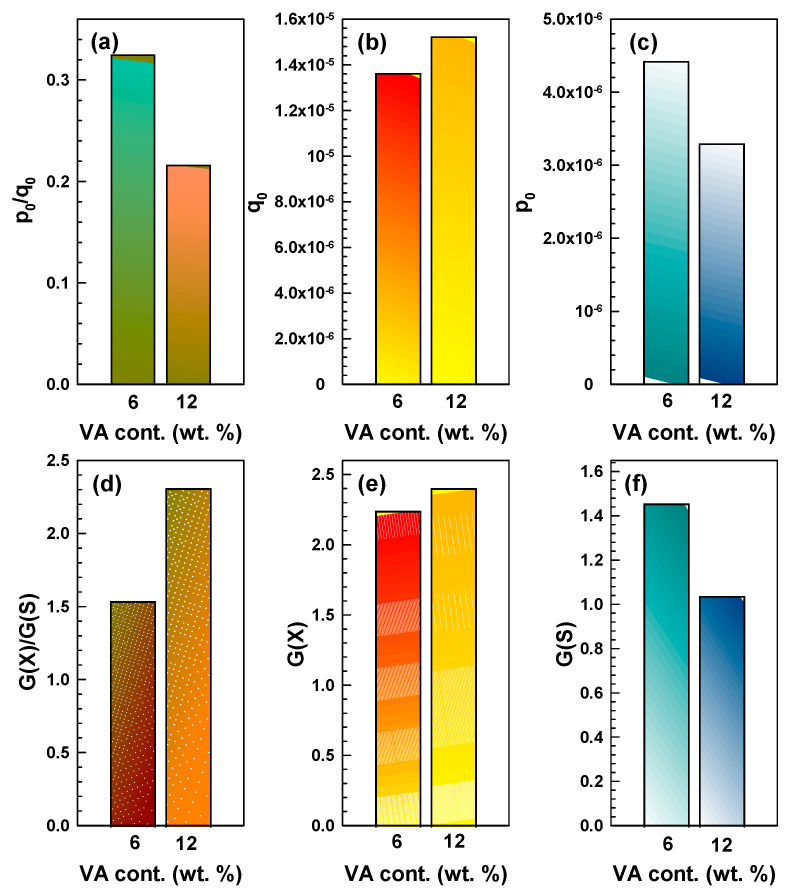
Results of the Charlesby–Pinner analysis: (**a**) $p_{0}/q_{0}$, (**b**) $q_{0}$, (**c**) $p_{0}$, (**d**) G(X)/G(S), (**e**) G(X), and (**f**) G(S).

In this case, we found that the $p_{0}/q_{0}$ ratio was lower for the copolymer with a higher content of VA (12 wt.%). When the copolymer content was 12 wt.%, the $p_{0}/q_{0}$ ratios ranged from 0.2, which suggests that the cross-linking processes predominated. Ramachandran et al. investigated the impact of radiation cross-linking on EOC-PDMS blends for cable insulation and reported lower $p_{0}/q_{0}$ values for the EOC, even at high radiation doses, indicating that cross-linking prevailed over chain scission [11]. They found out that in the case of EOC, even at increased radiation doses, the $p_{0}/q_{0}$ value is lower. This suggests that even at higher radiation doses, the cross-linking of the EOC chains prevails over the chain scission process. In Figure 14, the density of the cross-linked units per unit dose $q_{0}$ achieved a lower value for copolymer EVA 206 due to a lower amount of VA in the EVA copolymer. The fracture density per unit dose $p_{0}$ is lower for EVA 212 with a higher VA content. EVA 212 networks more efficiently due to a higher content of VA (12 wt.% of VA), resulting in a larger amount of amorphous phase that facilitates easier movement of radicals, which can react to each other, forming cross-links. The reduced chain scission in EVA 212 results in a higher cross-linking-to-scission ratio G(X)/G(S). This ratio was calculated using Equation (13):

(13) $$\frac{G\left(X\right)}{G\left(S\right)}=\frac{1}{2\frac{p_{0}}{q_{0}}}\;$$

Cross-linking and chain scission occurred simultaneously across the entire range of irradiation doses (0–180 kGy), with cross-linking appearing to be the dominant process. This observation aligns with the findings of Sabet and Soleimani [10]. Furthermore, the gel content measurements confirmed that the cross-linking capacity of the polymer samples increased with an increasing VA content.

**Scheme 2:**
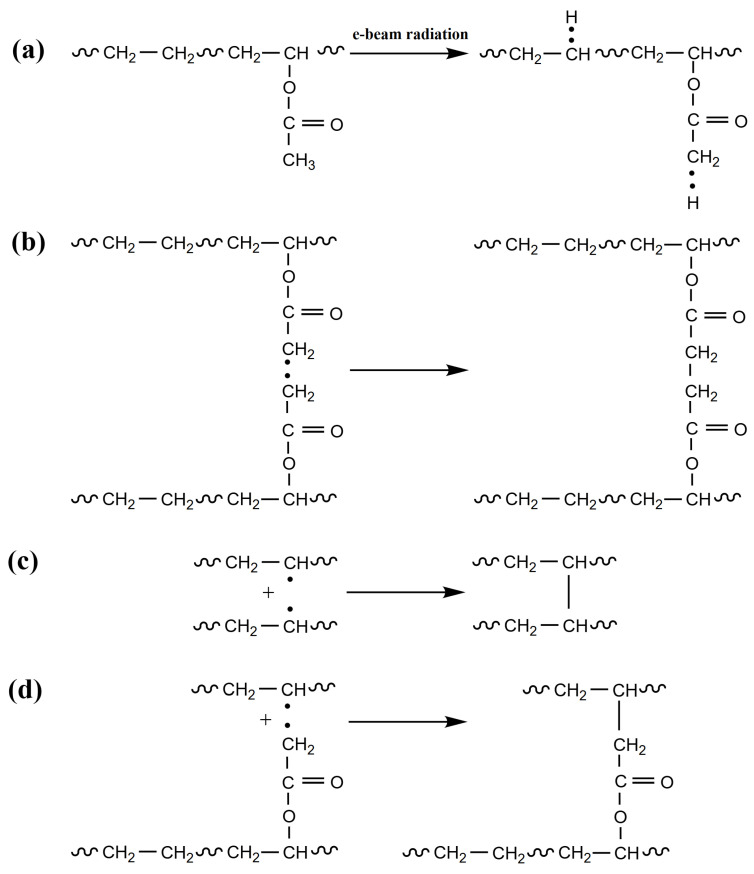
Possible cross-linking mechanism—chemistry: (**a**) generation of free radicals by irradiation; (**b**) cross-linking between vinyl acetate branches; (**c**) cross-linking between ethylene units; and (**d**) cross-linking between vinyl acetate branch and ethylene unit.

Scheme 2 illustrates a possible scheme of the cross-linking mechanism of EVA using electron beam radiation.

## 3. Materials and Methods

### 3.1. Materials

**Scheme 3:**
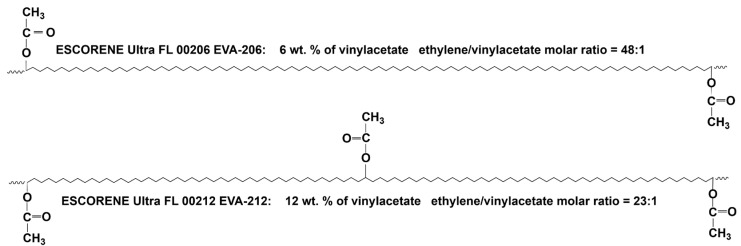
Chemical structure of the EVA copolymers.

Two distinct ethylene-vinyl acetate copolymers, commercially known as Escorene Ultra, were utilized in this study. These materials were provided by ExxonMobil Chemical (Houston, TX, USA). The copolymers contained 6 and 12 wt.% of vinyl acetate. For the purposes of this paper, these copolymers are designated as EVA 206 and EVA 212, respectively, based on their vinyl acetate content (wt.%). Table 6 provides a comprehensive overview of their compositions (both in weight and mole percentages) and densities. Both copolymers were intentionally selected to have identical initial melt flow indexes (MFI) of 2.0 g/10 min at 190 °C. Scheme 3 shows the possible structure of the EVA copolymers used in this study.

### 3.2. Compression Molding

Following ASTM D4703 [43], compression molding was performed to produce test sheets of 1.0 mm thickness. A stainless-steel frame measuring 12 cm × 6 cm was used to contain the material. Polymer pellets were preheated at 150 °C, and the preheating time was 5 min under minimal contact pressure. Subsequently, the material was compression molded at 10 MPa, the molding time was 3 min using a hydraulic press, and the molding temperature was 150 °C. The resulting sheets were then quenched under pressure using a separate cold press. The average cooling rate was 25 °C/min.

### 3.3. Electron Beam Irradiation

Electron beam irradiation was carried out at ambient temperature (25 °C) in air at BGS Beta-Gamma-Service GmbH, located in Wiehl, Germany. A Rhodotron toroidal electron accelerator (10 MeV, 200 kW) served as the radiation source. The irradiation process took place within a tunnel using a continuously moving conveyor system. The radiation dose was incrementally increased from 60 to 180 kGy, with each pass increasing the dose by 30 kGy, ensuring that the temperature remained below 50 °C. The samples were prepared in a single layer, sealed between PET sheets. Additional key parameters included a beam current of 10 mA, a conveyor belt speed of 3 m/min, a distance of 78 cm between the scanner and the samples, and an irradiation time of 2 s.

### 3.4. DMA Tests

**Figure 15:**
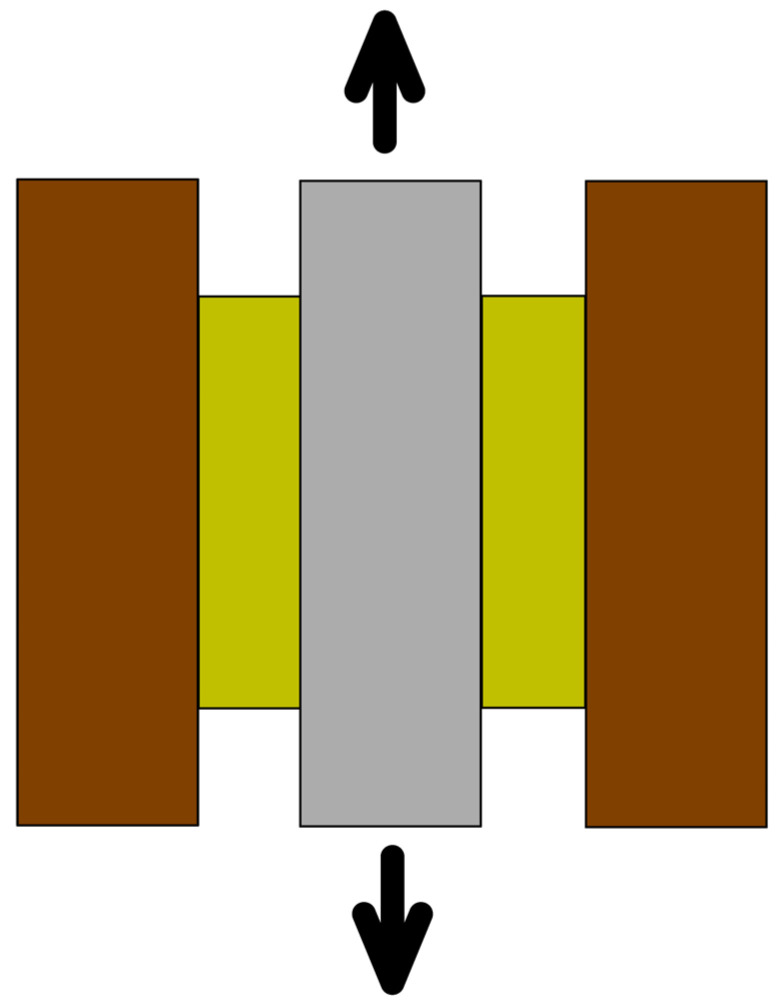
Shear mode of testing in Dynamic Mechanical Analyzer. The brown rectangles represent static metal pieces, the yellow-green rectangles represent measured samples (two pieces), and the gray rectangle is the central moving part where the black arrow shows the direction of movement. The movement of the central part (gray rectangle) is recorded. Many parameters, such as $G^{\prime },\;\eta^{*},\;\mathrm{and}\;\tan\delta$, are in a function of frequency in the range of 0.1–100 Hz.

**Figure 16:**
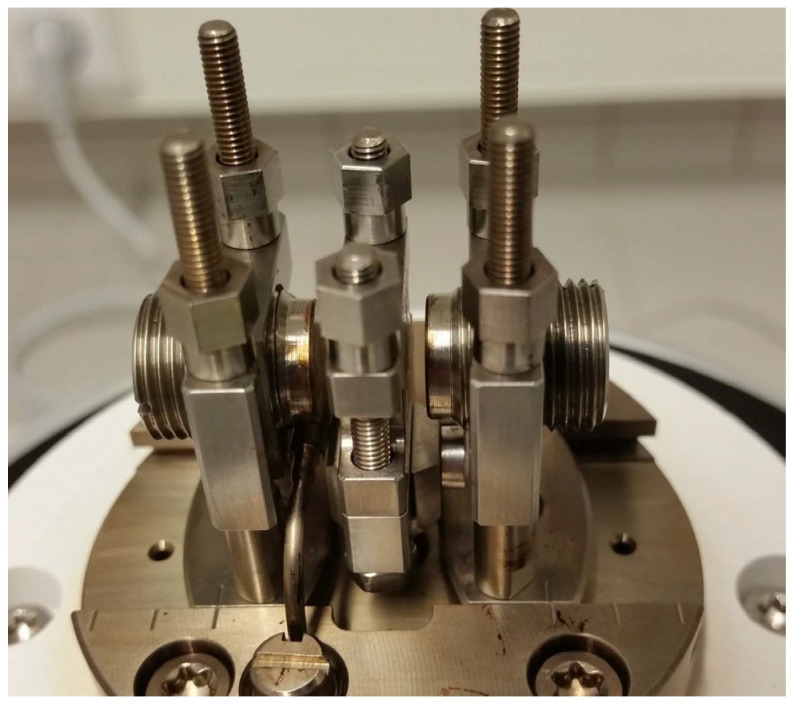
The actual testing equipment for shear mode testing in the Dynamic Mechanical Analyzer.

Dynamic mechanical analysis was conducted in a DMA-1 STARe System METTLER TOLEDO Switzerland machine (Mettler-Toledo (Switzerland) GmbH, Greifensee, Switzerland). All the tests in DMA were performed at an elevated temperature of 150 °C in shear mode, with two samples with a size of 11 × 11 × 1 mm. A frequency sweep was performed in DMA (Figure 15 and Figure 16). During the frequency sweep, the frequency was changed from 0.1 to 100 Hz. The force was 0.1 N, displacement was 10 μm, and the compared values were complex viscosity $\eta^{*}$, real part of shear storage modulus *G*′, and damping factor tanδ. The reported viscoelastic properties represent the the average of the measurements taken from three separate samples.

### 3.5. Gel Content

The gel contents of the electron beam cross-linked EVA 206 and EVA 212 samples were determined following ASTM D2765-01 [44]. This involved quantifying the insoluble fraction of the cross-linked material after solvent extraction. A 0.3 g sample was placed in a 120-mesh stainless-steel cage and extracted in refluxing xylene containing 1 wt.% of the antioxidant Irganox 1010 for 6 h. Post-extraction, the sample was dried in a vacuum oven at 55 °C for 24 h and subsequently weighed. The gel content was calculated as the ratio of the sample’s final weight (*m*_1_) to its initial weight (*m*_0_), expressed as a percentage. The reported gel content value represents the average of the measurements taken from three separate samples.

The gel content was calculated using the following formula:

(14) $$\mathrm{gel}\;\mathrm{content}\;\left(\%\right)=\frac{m_{1}}{m_{0}}\times 100\;$$

where $m_{1}$ is the sample weight after solvent extraction and $m_{0}$ is the initial sample weight [45].

### 3.6. Size-Exclusion Chromatography

Molecular weight measurements were performed at 160 °C using a Polymer Laboratories PL 220 high-temperature chromatograph (Polymer Laboratories, Varian Inc., Church Stretton, Shropshire, UK). The system was equipped with three 300 mm × 7.5 mm PLgel Olexis columns and a differential refractive index detector. 1,2,4-trichlorobenzene (TCB) served as the eluent, with a polymer concentration of 0.5 mg mL^−1^ that was stabilized by the antioxidant butylhydroxytoluene (BHT) (Ciba, Basel, Switzerland). A flow rate of 1 mL min^−1^ and a total volume of 200 mL were used for all samples. Calibration was performed using narrowly distributed polyethylene standards (Polymer Standards Service GmbH, Mainz, Germany).

### 3.7. Differential Scanning Calorimetry (DSC)

The melting point, crystallinity and glass transition temperatures were measured by differential scanning calorimeter DSC1 by Mettler Toledo, Columbus, OH, USA. Samples, approximately 10 mg in weight, were loaded into aluminum pans and measured with an empty pan serving as a reference. Measurements were conducted under inert nitrogen atmosphere at a gas flow rate of 200 mL/min.

### 3.8. Wide-Angle X-Ray Diffraction (WAXD)

WAXD was applied to obtain information about crystallinity. X-ray patterns were taken using an XRDynamic 500 diffractometer, Anton Paar, Graz, Austria, with Bragg-Brentano beam geometry in reflection mode, CuKα radiation with a Ni filter (λ = 0.154 nm) and the diffraction angle range of 2θ = 10–30° was used.

## 4. Conclusions

This study examined the effects of electron beam irradiation on the high-temperature dynamic mechanical properties of EVA copolymers, specifically those with 6 wt.% and 12 wt.% vinyl acetate (VA) contents, across irradiation doses of 60, 120, and 180 kGy. Using WAXD and DSC, it was established that EVA 212 (12 wt.% VA) exhibited lower crystallinity compared to EVA 206 (6 wt.% VA), leading to an increased amorphous phase that facilitated enhanced radical mobility and subsequent cross-link formation.

Consistent with previous research, the complex viscosity of the EVA copolymers increased with the radiation dose [24]. Moreover, a decrease in complex viscosity with increasing frequency was observed, confirming the pseudoplastic behavior of the irradiated materials [23].

Dynamic mechanical analysis revealed that a higher VA content resulted in a lower damping factor tanδ, indicating improved elasticity and increased cross-linking density. Furthermore, radiation exposure led to a reduction in tanδ and a concurrent increase in shear storage modulus $G^{\prime }$, reinforcing the correlation between irradiation and cross-linking.

The experimental findings conclusively demonstrate that EVA copolymers with a higher VA content exhibit a greater cross-linking density. This conclusion was independently validated through frequency sweep tests at 150 °C and a gel content analysis. The gel content measurements confirmed a higher degree of cross-linking in EVA 212, and the Charlesby–Pinner analysis showed an elevated cross-linking-to-scission ratio G(X)/G(S) for this copolymer.

Achieving optimal cross-linking density is critical for applications such as foam production. This study underscores the need for tailored investigations of each olefinic polymer to determine the most effective irradiation parameters [46].

## Figures and Tables

**Table 1:** The crystallinity of EVA copolymers measured by WAXD.

Sample	Crystallinity (%)
EVA 206–0 kGy	39.07
EVA 206–60 kGy	39.05
EVA 206–120 kGy	39.01
EVA 206–180 kGy	38.48
EVA 212–0 kGy	35.10
EVA 212–60 kGy	34.90
EVA 212–120 kGy	34.51
EVA 212–180 kGy	33.44

**Table 2:** Melting temperature $T_{m}$ and the degree of crystallinity X of the EVA copolymers measured by DSC.

Sample	Melting Temperature T_m_ (°C)	Degree of Crystallinity X (%)	Melting Temperature T_m_ (°C)	Degree of Crystallinity X (%)
EVA 206–0 kGy	102.38	43.75	102.09	43.64
EVA 206–60 kGy	99.19	41.46	98.78	42.00
EVA 206–120 kGy	98.95	40.15	97.82	38.60
EVA 206–180 kGy	98.60	39.54	96.80	36.76
EVA 212–0 kGy	95.93	35.58	96.11	37.73
EVA 212–60 kGy	93.63	33.93	92.87	34.17
EVA 212–120 kGy	92.95	33.65	90.92	33.67
EVA 212–180 kGy	92.75	33.41	89.92	33.01

**Table 3:** Shear thinning exponent (n) and (k) values from the Power law equation. (k) represents complex viscosity $\eta^{*}$ at frequency 1 Hz.

**Material**	**Dose (kGy)**	**n**	**k**
EVA 206	60 120 180	−0.75095 −0.83870 −0.89553	15,005 29,897 41,159
**Material**	**Dose (kGy)**	**n**	**k**
EVA 212	60 120 180	−0.77479 −0.85485 −0.91285	21,188 41,934 43,688

**Table 4:** Measured DMA data at 150 °C shear storage modulus $G^{\prime }$ at a frequency of 0.1 Hz for EVA 206. Calculated values of molecular weight between cross-links $M_{C}$ and cross-link density $\nu_{d}$.

Dose (kGy)	*G*′ (Pa)	M_c_ (kg/mol)	$\nu_{d}$ (mol/m^3^)
60	49,800	65.42	14.16
120	130,000	25.06	36.95
180	218,000	14.94	61.97

**Table 5:** Measured DMA data at 150 °C shear storage modulus $G^{\prime }$ at a frequency of 0.1 Hz for EVA 212. Calculated values of molecular weight between cross-links $M_{C}$ and cross-link density $\nu_{d}$.

Dose (kGy)	*G*′ (Pa)	M_c_ (kg/mol)	$\nu_{d}$ (mol/m^3^)
60	73,600	44.65	20.92
120	185,000	17.76	52.59
180	227,000	14.48	64.52

**Table 6:** Composition and density of the investigated ethylene-vinyl acetate copolymers (EVA).

Abbreviation	Trade Name	Vinylacetate (wt%)	Ethylene (wt%)	Vinylacetate (mol%)	Ethylene (mol%)	ET/VA (Molar Ratio)	Density (g/cm^3^)
EVA 206	Escorene Ultra FL 00206	6	94	2.038	97.962	48.1	0.926
EVA 212	Escorene Ultra FL00212	12	88	4.255	95.745	22.5	0.934

MFI at 190 °C was used for two samples, 2.0 g/10 min.

## Data Availability

The original contributions presented in this study are included in the article/Supplementary Materials. Further inquiries can be directed to the corresponding author(s).

## Supplementary Materials

The following supporting information can be downloaded at: https://www.mdpi.com/article/10.3390/molecules30071485/s1, Table S1: Dynamic oscillatory quantities from DMA testing; Table S2: Calculated Charlesby–Pinner parameters. Refs. [47,48,49,50,51,52,53,54,55] are cited in Supplementary Materials.
