# Supplementary material for: Dynamic Mechanical and Charlesby-Pinner Analyses of Radiation Cross-Linked Ethylene-Vinyl Acetate Copolymer (EVA)
Source: Molecules. 2025 Mar 27;30(7):1485. doi: 10.3390/molecules30071485 (PMC11990600; doi:10.3390/molecules30071485)
Supplement: Supplementary file 1 [file molecules-30-01485-s001.zip › molecules-3477816-supplementary.pdf]

Supplementary Material:

2. Theoretical background

Dynamic mechanical analysis as well as creep belong among the most used methods for description of viscoelastic behavior. These analyses focus on deep comprehension between structure and mechanical properties.

DMA – frequency sweep: the material response to increasing frequency (rate of deformation) is monitored at a constant amplitude and temperature.

G value: The G value represents the chemical yield of radiation, defined as the number of molecules reacting per 100 eV of absorbed energy. The unit eV is an old one, mol/J is used these days when SI units are preferred, the conversion ratio is this: 1/100 eV is approximately equal to 1.04×10^{-7} mol/J.

G (S) chain scission: This parameter quantifies the number of chain scissions occurring per 100 eV of absorbed energy.

G (X) cross-linking. This parameter quantifies the number of cross-links formed per 100 eV of absorbed energy.

Effects of electron beam ionizing method of radiolysis of polymers

There are two primary effects of radiation in polymers: cross-linking and chain scission. Different responses to radiation are observed among various polymers, particularly in terms of their tendencies toward either cross-linking or chain scission [1, 2]. Increasing radiation dosage leads to a higher cross-linking density, which can be observed through changes in the polymer's swelling behavior in a compatible solvent. Dominant cross-linking results in decreased swelling due to the increased network formation. Conversely, if chain scission is the predominant effect, increased swelling will be observed [3].

Polymers in which ionizing radiation causes cross-linking often have better mechanical properties [4]. Electron beam irradiation from accelerators is a common method for inducing cross-linking in polymer matrices [5]. However, the response of polymers to radiation varies; some undergo cross-linking, while others degrade. Polyethylene (PE), with its abundance of hydrogen atoms on side groups, exemplifies a polymer prone to radiation-induced cross-linking. Conversely, polymers containing methyl groups (e.g., polypropylene, PP), substitutions (e.g., polymethyl methacrylate, PMMA), or per-halogen substitutions (e.g., polytetrafluoroethylene, PTFE) are more likely to experience degradation. Aromatic polymers, such as polystyrene (PS) and polycarbonate (PC), exhibit greater radiation resistance due to the presence of benzene rings [6].

For polyolefins, the presence of secondary, tertiary, and quaternary carbons in the main-chain corresponds to an escalating likelihood of chain scission [7].

Typically, when any material is exposed to irradiation, both cross-linking and scission processes can occur concurrently. Nonetheless, it is common to notice a predominant occurrence of one process over the

other. Consequently, polymers can be classified into two groups: those that primarily undergo cross-linking and those that suffer from degradation. The application of high-energy radiation initiates a series of chemical reactions, resulting in either an increase or decrease in the molecular weight of the polymer [7].

Charlesby and Pinner [8] were the first to describe the relationship between the molecular weight of a polymer and the radiation-induced chain cross-linking.

Changes in polymer physical properties after irradiation are a consequence of the balance between cross-linking and chain scission. Radiation chemical yield is expressed using G values, specifically $G(X)$for cross-linking and $G(S)$ for chain scission. These values are often determined through sol-gel analysis using the Charlesby-Pinner equation.

Several forms of the Charlesby-Pinner equation exist. One common expression is

$$s + \sqrt{s} = \frac{p_0}{q_0} + \frac{1}{q_0 u_1 r} \qquad \text{(S1)}$$

where s represents the soluble polymer fraction, r is the radiation dose in kGy, q_0 is the cross-linked unit density per unit dose, p_0 is the ratio of main chain breaks to chain units, and $u_1$is the weight-average initial degree of polymerization.

The same equation is frequently expressed as [2]:

$$s + \sqrt{s} = \frac{p_0}{q_0} + \frac{1}{q_0 P_n D} \qquad \text{(S2)}$$

where D is the absorbed radiation dose in kGy and P_n is the initial number-averaged degree of polymerization.

A widely applied equation for analyzing the soluble fraction is [6]:

$$s + \sqrt{s} = \frac{G(S)}{2G(X)} + \frac{k_2}{G(X) M_n D} \qquad \text{(S3)}$$

where k_2 is equal to 4.82×10^6 when D is in kGy [7, 9]. $M_n$is the number-average molecular weight. The choice of application of Equation S3 or S4 is influenced by the mechanism of cross-linking [7]. Equation S3 is applicable under assumptions of a most probable initial molecular weight distribution, random scission and cross-linking, and H-linking cross-linking. For Y-linking cross-linking, the following equation is used:

$$s + \sqrt{s} = \frac{2G(S)}{G(X)} + \frac{k_3}{G(X) M_n D} \qquad \text{(S4)}$$

where k_3 is equal to 1.93×10^6 when D is in kGy [7, 9]. $M_n$is the number-average molecular weight.

Materials with a $G(S)$: $G(X)$ ratio less than 1 tend to cross-link, while those with a ratio greater than 1 tend to degrade. Materials with low $G(X)$ and $G(S)$ values exhibit higher radiation resistance [6].

Turgis et al. [10] proposed a modified equation for AB copolymers:

$$s + \sqrt{s} = \frac{G(S)}{2G(X)} + \frac{9.65 \cdot 10^6 \cdot m_A}{G(X) \cdot D \cdot M_w \cdot [x_B \cdot m_B + (1 - x_B) \cdot m_A]} \quad \text{(S5)}$$

Where m_A and m_B are molecular weights of comonomer units A and B, respectively. x_B is the molar fraction of comonomer B.

Power law theory

This study investigates the rheological properties of irradiated ethylene copolymers using dynamic mechanical test data. The method involves subjecting the sample to sinusoidal deformation for several cycles in dynamic mode and analyzing the resulting stress response.

Stress oscillates sinusoidally at the same frequency as the strain wave, but with a phase angle shift δ. This can be mathematically expressed as shown in Table S1:

Table S1. Dynamic oscillatory quantities from DMA testing

DMA Parameter	Symbol	Equation
Shear strain	$\gamma(t)$	$\gamma(t) = \gamma_0 \cdot sin\,\omega t$<br>$\gamma = \gamma_0 \cdot sin(\omega t + \delta)$
Shear strain amplitude	γ_0	$\gamma_0 = \frac{\Delta_0}{h}$
Rate of shear strain	$\dot{\gamma}$	$\dot{\gamma} = \frac{d\gamma}{dt}$
Shear stress	τ	$\tau = \tau_0 \cdot sin(\omega t + \delta)$
Complex shear modulus	G^*	$G^*(\omega) = \frac{\tau_0}{\gamma_0} = G' + iG''$
Storage shear modulus	$G'(\omega)$	$G'(\omega) = \frac{\tau_0}{\gamma_0} cos\,\delta$
Loss storage modulus	$G''(\omega)$	$G''(\omega) = \frac{\tau_0}{\gamma_0} sin\,\delta$
Complex viscosity	$\eta^*(\omega)$	$\eta^*(\omega) = \frac{\tau^*}{\gamma^*} = \eta' + i\eta''$
Elastic component	$\eta'(\omega)$	$\eta'(\omega) = \frac{\tau_0}{\omega\gamma_0} sin\,\delta = \frac{G''}{\omega}$
Viscous component	$\eta''(\omega)$	$\eta''(\omega) = \frac{\tau_0}{\omega\gamma_0} cos\,\delta = \frac{G'}{\omega}$
Damping factor	$tan\,\delta$	$tan\,\delta = \frac{G''(\omega)}{G'(\omega)} = \frac{\eta'(\omega)}{\eta''(\omega)}$

For the frequency sweep data, the power law expression relates complex viscosity to frequency:

$$|\eta^{*}| = k\omega^{n} \qquad \text{(S6)}$$

where k is a material-specific pre-exponential factor, ω is the frequency, and n is the shear thinning exponent [11]. Shear thinning is often observed in cross-linked polymers and polymer composites [12]. When the index n has a negative value, the effective viscosity decreases as the frequency increases. Consequently, this model represents the disintegration of fluid structure under shear, commonly known as the shear thinning [13].

The parameters k and n can be determined from the logarithmic plot of complex viscosity versus frequency [11]

$$log|\eta^{*}| = log\, k + n\, log\, \omega \qquad \text{(S7)}$$

Valles et al. claims that the power law exponent declines with increasing radiation dose (and thus increasing cross-linking). This observation is supported by independent measurements of gel content and molecular weight distribution [14].

Rheological properties are strongly influenced by molecular weight and structural features like branching and cross-linking [15].

Appendix

Calculation of Charlesby–Pinner parameters for EVA 206

6 wt % of vinyl acetate, M_n = 39000 g/mol

$$\text{ethylene } = -CH_2-CH_2-, M_{ET} = 2 \cdot C + 4 \cdot H = 2 \cdot 12.011 + 4 \cdot 1.008 = 28.054\ g/mol$$

$$\text{vinyl acetate } = C_4H_6O_2, M_{VA} = 4 \cdot C + 6 \cdot H\ + 2 \cdot O = 4 \cdot 12.011 + 6 \cdot 1.008 + 2 \cdot 16 = 86.092\ g/mol$$

$$\text{wt. fraction of vinyl acetate } w_{VA} = \frac{6}{100} = 0.06$$

$$\text{wt. fraction of ethylene } w_{ET} = 1 - w_{VA} = 1 - 0.06 = 0.94$$

$$\text{Molar fraction of vinyl acetate } = x_{VA}$$

$$x_{VA} = \frac{\frac{w_{VA}}{M_{VA}}}{\frac{w_{VA}}{M_{VA}} + \frac{w_{ET}}{M_{ET}}} = \frac{\frac{0.06}{86.092}}{\frac{0.06}{86.092} + \frac{0.94}{28.054}} = 0.02038$$

$$\text{Molar fraction of ethylene } = x_{ET} = 1 - x_{VA} = 1 - 0{,}020378 = 0.97962$$

Average molecular weight of repeating unit:

$$M_{ET-VA} = x_{ET}M_{ET} + x_{VA}M_{VA} = 0.97962 \cdot 28.054 + 0.02038 \cdot 86.092$$
$$= 29.2368\ g/mol$$

$$\text{Polymerization degree } = P_n = \frac{M_{nVA}}{M_{ET-VA}} = \frac{39000}{29.2368} = 1334$$

Charlesby–Pinner equation:

$$s + \sqrt{s} = \frac{p_0}{q_0} + \frac{1}{q_0 P_n D}$$

$$\text{In plot } s + \sqrt{s}\ vs. \frac{1}{D} : intercept = \frac{p_0}{q_0}, slope\ \frac{1}{q_0 P_n}$$

In case of EVA 206: intercept = 0.3246, slope = 55.072

$$\text{then } \frac{p_0}{q_0} = 0.3246 \text{ and } \frac{1}{q_0 P_n} = 55.072$$

$$q_0 = \frac{1}{slope \cdot P_n} = \frac{1}{55.072 \cdot 1334} = 0.00001361$$

$$\text{then } p_0 = q_0 \cdot intercept = q_0 \cdot \frac{p_0}{q_0} = 0.00001361 \cdot 0.3246 = 0.000004418$$

Calculation of G parameters according to Charlesby-Pinner equation (see Table S2):

$$s + \sqrt{s} = \frac{G(S)}{2G(X)} + \frac{4.82 \times 10^6}{G(X)M_n D}$$

$$\text{then } \frac{G(S)}{2G(X)} = \frac{p_0}{q_0}$$

$$\frac{G(X)}{G(S)} = \frac{1}{2\frac{p_0}{q_0}} = \frac{1}{2 \cdot 0.3246} = 1.5404$$

$$\text{slope } = \frac{4.82 \times 10^6}{G(X)M_n}$$

$$G(X) = \frac{4.82 \times 10^6}{slope\ \cdot\ M_n} = \frac{4.82 \times 10^6}{55.072 \cdot 39000} = 2.2441$$

$$\text{Intercept} = \frac{G(S)}{2G(X)}$$

$$G(S) = 2 \cdot G(X) \cdot intercept = 2 \cdot 2.2441 \cdot 0.3246 = 1.4569$$

Calculation of Charlesby–Pinner parameters for EVA 212

12 wt % of vinyl acetate, M_n = 34000 g/mol

$$\text{ethylene} = -CH_2-CH_2-, M_{ET} = 2 \cdot C + 4 \cdot H = 2 \cdot 12.011 + 4 \cdot 1.008 = 28.054\ g/mol$$

$$\text{Vinyl acetate} = C_4H_6O_2, M_{VA} = 4 \cdot C + 6 \cdot H + 2 \cdot O = 4 \cdot 12.011 + 6 \cdot 1.008 + 2 \cdot 16 = 86.092\ g/mol$$

$$\text{wt. fraction of vinyl acetate } w_{VA} = \frac{12}{100} = 0.12$$

$$\text{wt. fraction of ethylene } w_{ET} = 1 - w_{VA} = 1 - 0{,}12 = 0.88$$

$$\text{Molar fraction of vinyl acetate} = x_{VA}$$

$$x_{VA} = \frac{\frac{w_{VA}}{M_{VA}}}{\frac{w_{VA}}{M_{VA}} + \frac{w_{ET}}{M_{ET}}} = \frac{\frac{0.12}{86.092}}{\frac{0.12}{86.092} + \frac{0.88}{28.054}} = 0.04255.$$

$$\text{Molar fraction of ethylene} = x_{ET} = 1 - x_{VA} = 1 - 0.04255 = 0.95745$$

Average molecular weight of repeating unit:

$$M_{ET-VA} = x_{ET}M_{ET} + x_{VA}M_{VA} = 0.95745 \cdot 28.054 + 0.04255 \cdot 86.092 = 30.5235\ g/mol$$

$$\text{Polymerization degree} = P_n = \frac{M_{nVA}}{M_{ET-VA}} = \frac{34000}{30.5235} = 1114$$

Charlesby–Pinner equation:

$$s + \sqrt{s} = \frac{p_0}{q_0} + \frac{1}{q_0 P_n D}$$

$$\text{In plot } s + \sqrt{s}\ vs. \frac{1}{D}: intercept = \frac{p_0}{q_0}, slope\ \frac{1}{q_0 P_n}$$

In case of EVA 212: intercept = 0.2163, slope =58.972

$$\text{then} \frac{p_0}{q_0} = 0.2163\ and\ \frac{1}{q_0 P_n} = 58.972$$

$$q_0 = \frac{1}{slope \cdot P_n} = \frac{1}{58.972 \cdot 1114} = 0.00001522$$

$$\text{then } p_0 = q_0 \cdot intercept = q_0 \cdot \frac{p_0}{q_0} = 0.00001522 \cdot 0.2163 = 0.000003292.$$

Calculation of G parameters according to Charlesby-Pinner equation (see Table S2):

$$s + \sqrt{s} = \frac{G(S)}{2G(X)} + \frac{4.82 \times 10^6}{G(X)M_n D}$$

$$\text{then } \frac{G(S)}{2G(X)} = \frac{p_0}{q_0}$$

$$\frac{G(X)}{G(S)} = \frac{1}{2\frac{p_0}{q_0}} = \frac{1}{2 \cdot 0.2163} = 2.3116$$

$$\text{slope} = \frac{4.82 \times 10^6}{G(X)M_n}$$

$$G(X) = \frac{4.82 \times 10^6}{58.972 \cdot 34000} = 2.4039$$

$$\text{Intercept} = \frac{G(S)}{2G(X)}$$

$$G(S) = 2 \cdot G(X) \cdot intercept = 2 \cdot 2.4039 \cdot 0.2163 = 1.0399$$

Table S2. Calculated Charlesby–Pinner parameters.

**Material**	**wt% of VA**	**wt.fraction of VA**	**wt.fraction of ET**	**Molar fraction of VA**
EVA206	6	0.06	0.94	0.0204
EVA212	12	0.12	0.88	0.0425
**Material**	**Molar fraction of ET**	**M_{ET-VA}**	**M_n**	**P_n**
EVA206	0.9796	29.2366	39000	1333.95
EVA212	0.9575	30.5232	34000	1113.91
**Material**	**Slope**	**p_0/q_0**	**q_0**	**p_0**
EVA206	55.0716	0.3246	0.00001361	0.000004418
EVA212	58.9716	0.2163	0.00001522	0.000003293
**Material**	**G(X)/G(S)**	**G(X)**	**G(S)**	
EVA206	1.5405	2.2442	1.4568	
EVA212	2.3116	2.404	1.0399	

References

[1] Clegg DW. Irradiation effects on polymers. London, United Kingdom: Elsevier Applied Science; 1991.

[2] Svoboda P. High-temperature study of radiation cross-linked ethylene-octene copolymers. Polymer Bulletin. 2017;74(1):121-144. https://doi.org/10.1007/s00289-016-1703-6.

[3] Ghobashy M. Ionizing Radiation-Induced Polymerization. Rijeka, Croatia: IntechOpen; 2018.

[4] Chmielewski AG. Applications of ionizing radiation in materials processing. Warszawa, Poland: Institute of Nuclear Chemistry and Technology; 2017.

[5] Datta S, Naskar K, Bhardwaj YK, Sabharwal S. A study on dynamic rheological characterisation of electron beam crosslinked high vinyl styrene butadiene styrene block copolymer. Polymer Bulletin. 2011;66(5):637-647. https://doi.org/10.1007/s00289-010-0359-x.

[6] Makuuchi K, Cheng S. Radiation Processing of Polymer Materials and Its Industrial Applications. Hoboken, New Jersey: John Wiley & Sons, Inc.; 2012.

[7] Hill DJT, Whittaker AK. Radiation Chemistry of Polymers. Encyclopedia of Polymer Science and Technology. Hoboken, New Jersey: John Wiley & Sons, Inc.; 2004.

[8] Charlesby A, Pinner SH. Analysis of the Solubility Behaviour of Irradiated Polyethylene and Other Polymers. Proceedings of the Royal Society of London Series a-Mathematical and Physical Sciences. 1959;249(1258):367-386. https://doi.org/10.1098/rspa.1959.0030.

[9] Thomas J, Thomas S, Ahmad Z. Crosslinkable Polyethylene: Manufacture, Properties, Recycling, and Applications. Singapore: Springer Nature 2021.

[10] Turgis JD, Coqueret X. Electron beam sensitivity of butyl acrylate copolymers: effects of composition on reactivity. Macromolecular Chemistry and Physics. 1999;200(3):652-660. https://doi.org/10.1002/(Sici)1521-3935(19990301)200:3<652::Aid-Macp652>3.0.Co;2-4.

[11] Durmuş A, Woo M, Kaşgöz A, Macosko CW, Tsapatsis M. Intercalated linear low density polyethylene (LLDPE)/clay nanocomposites prepared with oxidized polyethylene as a new type compatibilizer: Structural, mechanical and barrier properties. European Polymer Journal. 2007;43(9):3737-3749. https://doi.org/10.1016/j.eurpolymj.2007.06.019.

[12] Dutta J, Ramachandran P, Ismail SMRS, Naskar K. Melt Rheological Behavior and Creep Response of EVA/TPU Blends: Exploring the Effect of Electron Beam Irradiation and Peroxide Cross-linking. Polymer-Plastics Technology and Engineering. 2017;56(4):421-434. https://doi.org/10.1080/03602559.2016.1227843.

[13] Balmforth N, Craster R. Geophysical Aspects of Non-Newtonian Fluid Mechanics. Berlin, Germany: Springer-Verlag Berlin Heidelberg 2001.

[14] Vallés EM, Carella JM, Winter HH, Baumgaertel M. Gelation of a radiation crosslinked model polyethylene. Rheologica Acta. 1990;29(6):535-542. https://doi.org/10.1007/BF01329300.

[15] Shin BY, Ha MH, Han DH. Morphological, Rheological, and Mechanical Properties of Polyamide 6/Polypropylene Blends Compatibilized by Electron-Beam Irradiation in the Presence of a Reactive Agent. 2016;9(5):342. https://doi.org/10.3390/ma9050342.
